# Production of the *Bacillus licheniformis* SubC protease using *Lactococcus lactis* NICE expression system

**DOI:** 10.1186/2193-1801-1-54

**Published:** 2012-11-29

**Authors:** Aleksandra M Mirończuk, Anna Krasowska, Anna Murzyn, Małgorzata Płachetka, Marcin Łukaszewicz

**Affiliations:** 1 Department of Biotechnology and Food Microbiology, Wrocław University of Environmental and Life Sciences, Chełmońskiego 37/41, Wrocław, 51-630 Poland; 2 Department of Biotransformation, Faculty of Biotechnology, University of Wroclaw, Przybyszewskiego 63-77, Wroclaw, 51-148 Poland; 3 Faculty of Chemistry, Wrocław University of Technology, Gdańska 7/9, Wrocław, 50-344 Poland

**Keywords:** Lactic acid bacteria, *Lactococcus lactis*, Nisin-controlled expression system, NICE, *Bacillus licheniformis*, SubC protease

## Abstract

In this work the *subC* gene from *Bacillus licheniformis* encoding subtilisin was cloned into the nisin-controlled expression (NICE) vectors (pNZ8048 and pNZ8148) with or without the signal peptide SP Usp45 directing extracellular secretion via Sec machinery. Extracellular protease production and activity was tested using *Lactococcus lactis* NZ9000 as host, which could be used for rennet production. The efficiency of protein production was tested using purified nisin and the supernatant of *L. lactis* NZ970 nisin producer. Similar results were obtained for 1 ng/ml nisin and 10 000 diluted supernatant. SP Usp45 signal peptide effectively directed extracellular localization of active and stable protease. SubC signal for extracellular localization in *B. licheniformis*, was also recognized by *L. lactis* Sec pathway, although with lower efficiency, as shown by a 3-fold lower protease activity in the medium. Protease production and activity was optimized using parameters such as induction time, nutrients (glucose, casitone) supplementation during growth or protease stabilization by calcium ions. The results were also verified in fed-batch bioreactor for further scale-up of the expression system.

## Introduction

*Lactococcus lactis* is a Gram-positive, lactic acid bacterium that is commonly used in traditional food industries such as in cheese and butter production. In addition, it is increasingly used in modern biotechnological applications. Many recent studies have investigated the physiology and genetic of this bacterium, therefore a wide variety of genetic tools have been developed. Nowadays several genomes of *L. lactis* strains are completely sequenced (Bolotin et al. 2001; Siezen et al. 2010; Wegmann et al. 2007). Genetic accessibility and the ease of working with this organism have led to extensive study on heterologous protein expression in *L. lactis*. Since *L. lactis* is generally recognized as safe (GRAS) it could be used for large-scale production of heterologous proteins (Mierau et al. 2005a; Morello et al. 2008). Most of the laboratory scale examples consist of intracellular expression (Blatny et al. 2003; Kunji et al. 2003) or cell wall bound enzymes (Cibik et al. 2001; Miyoshi et al. 2002; Nouaille et al. 2003). Much less in known about extracellular production of the proteins in *L. lactis*, moreover the protein yield might differ significantly and are strongly case-dependent (Mierau & Kleerebezem 2005).

In 1995 Kuipers et al. (1995) published a study on the autoinduction of the expression of nisin in *lactococci*. This study allowed for the construction of a food grade expression system based on the regulation mechanism of the nisinA operon of *L. lactis* (Platteeuw et al. 1996), named NICE (nisin-controled gene expression). In this operon the gene product, a small 34 amino acid bacteriocin, induces its own transcription at very low concentrations (0.5 – 5 ng/mL) (Kuipers et al. 1998). In this system, nisin induces the regulatory cascade starting with binding to the membrane-bound receptor NisK. Next, the phosphate group from the activated NisK is transferred to the intracellular response regulator NisR, activating this regulator. Subsequently, NisR, induces the nisin operon at the promoter nisA (Kleerebezem & Quadri 2001). The NisA promoter controls the expression of the genes involved in the nisin biosynthesis (or the gene of interest). Genes of this regulatory system have been inserted in a suitable host strain *L. lactis* NZ900. The nisin-producing strain *L. lactis* NZ9700 secretes the nisin into the medium (Kuipers et al. 1995).

It was shown that the NICE system can be developed to “food-grade” production of heterologous protein, by replacing the antibiotic resistant gene by another selectable marker (Olempska-Beer et al. 2006), for example *lacF*, which has been deleted from the host strain, and is essential for growth on lactose (Mierau et al. 2005b). Although for large-scale production nisin usage remains costly, a good alternative is the addition of NZ9700 supernatant. The NICE system is often used in the laboratories for research, while the data on large-scale application of the NICE system for secreted proteins is still very limited. Up to now, only a few reports present usage of this system in industry (Mierau et al. 2005a; Mierau et al. 2005b; Berlec & Strukelj 2009), moreover, efficient systems for the industrial scale production of secrete heterologous proteins have never been described.

In this study, we describe the production of secrete heterologous protein SubC in *L. lactis* using the NICE expression system. SubC is the industrially important Carlsberg-type subtilisin (Jacobs 1995) produced by *Bacillus licheniformis*. Bacterial subtilisins are multipurpose alkaline proteases that are frequently used in industry (Gupta et al. 2002): common variants include subtilisin from *B. amyloliquefaciens*, highly alkalophilic *B. lentus* or from *B. licheniformis* (Rao et al. 1998; von der Osten et al. 1993). Furthermore, *Flavobacterium* also produces subtilisin (Morita et al. 1998). Interestingly, almost two-third of commercial proteases produced in the world originate from microorganisms (Kumar & Takagi 1999; Tremacoldi et al. 2004). Microbial proteases are classified into different groups, according to their activity in acid, neutral or alkaline conditions, and on the characteristics of the active site group of the enzyme (Saeki et al. 2007). Subtilisin-like serine proteases are usually secreted extracellulary for searching nutrients (Aehle et al. 2009). One of the features of this class of proteases (subtilases) is an aromatic or hydrophobic residue, such as leucine, tyrosine or phenylalanine. The highest proteolytic activity is around pH 10, with a molecular weight range of 15–40 kDa and an isoelectric point around pI 9. Moreover, the native form of SubC remains fully stable up to 60°C (Hirata et al. 2003) and it possesses two calcium binding site(s), therefore bound Ca^2+^ contributes to enzyme stability (Briedigkeit & Frömmel 1989).

Our main objective in this study was an improvement of the expression condition for secreted heterologous protein production and the comparison of the gene expression efficiencies using two different NICE expression plasmids. We compared two different NICE vectors and the ability of *L. lactis* for secretion of heterologous protein. The activity of protease production was tested on milk plates. Subsequently the proteolytic activity assays were performed to investigate the functionality of the secreted protease in the medium. In addition, we present modification of the NICE system, by changing the growth conditions, the induction point, and by extending the logarithmic phase growth of bacteria by supplying further nutrients.

## Methods

### Strains and growth conditions

The strains and plasmids used in this study are listed in Table 1.

**Table 1:** **Strains used in this study**

***Strain/plasmid***	***Relevant characteristics***	***Reference***
ATCC 10716	*B. licheniformis*	Laboratory stock
NZ9000 *L. lactis*	MG1363 *pepN*::nisRK	(Kuipers et al. 1998)
NZ9700 *L. lactis*	Nisin producer	(Kuipers et al. 1995)
pNZ8048	Cm^R^, inducible expression vector containing the *nisA* promoter	(Kuipers et al. 1998)
pNZ8148	PnisA, CmR; replicon of rolling circle plasmid pSH71, basic NICE vector, derivative of pNZ8048	NIZO
pNZ45	pNZ8048 carrying SP Usp45 under *nisA* promoter	This study
pNZ45subC	pNZ45 carrying *subC* gene	This study
pNZ48subC	pNZ8148 carrying *subC* gene	This study

*Lactococcus lactis* strains were grown in M17 broth (Terzaghi & Sandine 1975) supplemented with 0.5% glucose (GM17). Additionally, for strains carrying plasmid, medium was supplemented with chloramphenicol (5 μg mL^-1^). Cultures were incubated at 30°C. If required, medium was supplemented with 10% milk or different concentrations of CaCl_2_, MnCl_2_, MgCl_2_ and MgSO_4_. Additionally, samples were supplemented with 5 μg mL^-1^ chloramphenicol, if required. Bacterial growth was determined by measuring the optical density (OD) at 600 nm. The cultures were inoculated at optical density 0.1 or at a different point if mentioned. At the beginning of the incubation or when the bacterial growth reached the required cell density, the cultures were stimulated to produce protease by the addition of nisin (Sigma) or by the dilutions of the culture supernatant of the nisin producing strain *L. lactis* NZ9700 (the range of concentrations or dilutions indicated in Results). The batch cultivations were performed in a 5-l stirred-tank reactor (Labfors 3; Infors, Swizerland) with a working volume of 1.5 l at 30°C. The stirrer speed was adjusted to 60 rpm and the pH was maintained automatically at 7.0 by the addition of 1M NH_4_OH. The cultures were grown in the M17 medium supplemented with 10% milk, 0.5% glucose and chloramphenicol (5 μg mL^-1^); induction at optical density 0.2. The samples were taken every 2 hours.

### Construction of pNZ45subC, pNZ48subC

It was shown, that Usp45 signal peptide of *L. lactis* enables secretion of the heterologous protein via the Sec pathway (Morello et al. 2008) in *L. lactis* strains (van Asseldonk et al. 1993). Therefore, to express the secretion protein, first the *usp45* peptide signal was amplified with Usp-45-F (5’-GCGC CCATGGGGAAAAAAAAGATTATCTCAGCTATTTTAATG-3’) and Ups-45-R (5’-CGCCGCATGCCCCGGGTGTGT CAGCGTAAAC-3’) primers. Subsequently the 120 bp PCR product was cloned into the *NcoI*/*SphI* sites of pNZ8040 (Kuipers et al. 1998), resulting in pNZ45. After amplification of *B. licheniformis* subC gene with subC- F (5’-ATGAGGAAAAA GAGTTTTTG-3’) and subC- R (5’-CGCGTCTAGATTATTGAGCGGCAGCTTC-3’) primers, the 1139 bp PCR fragment was digested with *XbaI* and cloned into the pNZ45 digested with *SmaI* and *XbaI*, yielding in pNZ45subC. The SP Usp45 fragment is supposed to be cleaved during secretion of protein via Sec translocon into the environment (Mierau & Kleerebezem 2005). Finally, for the control, subC gene was fused translationally to the Nisin-inducible promoter P_nisA_ of pNZ8148 (lack of SP Usp45). To overexpress SubC in pNZ8148 (Mierau & Kleerebezem 2005), the gene was amplified with subC-48-F (5’-GCCGCCATGGGGATGATGAGGAAAAAGAG-3’) and subC-48-R (5’-CGCGGCATGCTTATTGAGCGGCAGCTTC-3’) primers. The PCR was digested with *NcoI* and *SphI* and ligated into the corresponding sites of pNZ8148, resulting in pNZ48subC. Transformation of *L. lactis* NZ9000 was achieved by electroporation using Gene pulser (Biorand Laboratories) as described by Leenhouts an Venema (Leenhouts & Venema 1993).

### Expression of SubC in *L. lactis* and sample preparation

*L. lactis* NZ900 carrying pNZ45subC or pNZ48subC was cultivated in M17 medium (Difco) containing 0.5% glucose and 5 μg mL^-1^ chloramphenicol. To test for the expression of SubC *L. lactis* was grown in 10 ml cultures, diluted from overnight culture to an OD_600_ of 0.1 at 30°C. The samples were induced at start point with NisinA (1:10000 dilution of the culture supernatant of the nisin producing strain *L. lactis* NZ9700) and the cultures were incubated for 24 hours. 1.5 mL of culture was spun down (14000 rpm, 10 min, 4°C) and 500 μL of supernatant was transferred into a fresh tube. Next, the same volume of cold 10% TCA (trichloroacetic acid) was added. The sample was incubated for 5 min at −20°C and subsequently was spun down (14000 rpm, 10 min, 4°C) then the supernatant was discarded. The pellet was washed with 500 μL of cold acetone and spun down under the same conditions. The pellet was resuspended in 20 μL PBS buffer (137 mMol/L NaCl; 1.76 mMol/L KH_2_PO_4_; 8.1 mMol/L Na_2_HPO4 x H_2_O; 2.7 mMol/L KCl). The effect of heterologous protein expression in differently supplemented M17 medium, was compared by 12% sodium dodecyl sulfate-polyacrymide gel electrophoresis (SDS-PAGE) followed by Coomassie staining. The samples were diluted 2x SDS sample buffer and aliquots of 10 μl were loaded per lane. Molecular masses were estimated using PageRuler™ Prestained Protein Ladder (Fermentas).

### Proteolytic activity assay

Proteolitic activity was assayed using 0.5% casein (Sigma) as a substrate, dissolved in 50 mM Tris–HCl buffer at pH 10. Tests were performed in final volume of 800 μL containing 500 μL 0.5% casein, 200 μL 50 mM Tris–HCl buffer pH 10 and 100 μL of supernatant from the tested culture. Samples were incubated at 50°C for 30 min. Reaction was stopped by the addition of 500 μL of 10% trichloroacetic acid. Control samples were assayed similarly but the supernatants were added after the addition of trichloroacetic acid. Samples were centrifuged for 10 min at 15 000 rpm at 4°C. The absorbance of free tyrosine was measured at 275 nm using Thermo Electron Corporation Evolution 600 UV-Visible Spectrophotometer. To calculate the proteases activity, an extinction curve for tyrosine was prepared. Various concentrations of tyrosine in 50 mM Tris buffer at pH 10 were incubated as treated samples. One unit of enzyme activity was defined as the amount of enzyme that releases 1 μg tyrosine per 1 min under these conditions.

### Amino acid sequence analysis

Proteins from the culture supernatant were salted out using 50% ammonium sulfate and visualized by SDS-PAGE gel electrophoresis. Then, proteins were transferred on the PVDF membrane and the 38-kDa band (containing subtilisin C) was chosen for sequencing. Amino acid sequencing was performed by using Edman degradation.

## Results

### Overexpression of SubC in *L. lactis* by nisinA or supernatant of NZ9700

In this report, two standard vectors, pNZ8148 (Kumar & Takagi 1999) and pNZ8048 (Mierau et al. 2005b), were used for direct expression of *B. licheniformis* SubC protein (Hirata et al. 2003). Two constructs pNZ45subC or pNZ48subC (for details see Material and Methods) were introduced into NZ900 *L. lactis* strain. For the induction of heterologous protease, various growth conditions such as the induction time point and the range of nisin concentrations have been investigated. The activity of the supernatant of pNZ45subC or pNZ48subC was tested against casein substrate (see material and methods). Casein was used for proteolytic assays, since casein is the most frequently used substrate for protease activity under neutral and alkaline conditions.

Initially tests were performed on the GM17 plates supplemented with 10% milk, consequently the same conditions were used during growth in liquid medium. The samples were induced at the beginning of growth at the optical density 0.1. Protein expression was induced by the addition of nisinA in the final concentration of 0.1 ng mL^-1^, 0.5 ng mL^-1^, 1 ng mL^-1^, 2 ng mL^-1^, 5 ng mL^-1^ and 10 ng mL^-1^. The induced and non-induced cells were harvested after 24 h, and the proteolytic activity assay was performed. The results are listed in Table 2. The highest proteolytic activity was observed after induction of 1 ng/mL, in all non-induced strains proteolytic activity was close to zero. To verify the optimal conditions for protein expression, a range of nisin and the NZ9700 supernatant concentrations was tested (100 × dilution to 200 000 × dilution of the culture supernatant of the nisin producing strain *L. lactis* NZ9700). The results are listed in Table 3. All induced cultures showed different ranges of enzymatic activity; in all non-induced cultures the values of proteolytic activity was below 1. The highest proteolytic activity was obtained when 10 000 – 20 000 diluted supernatant of *L. lactis* NZ9700 was used. This data showed that SubC was secreted into the growth medium and the protein remained active. Similar activity was observed when cultures were induced with 1 ng mL^-1^ purified nisinA (see Table 2) or 10 000 – 20 000 diluted supernatant of NZ9700. Since the use of purified nisinA at industry scale might be costly, in further experiments for the induction the supernatant of the NZ9700 strain was added. The samples were collected for the proteolytic assays after 24 hours after induction.

**Table 2:** **The proteolytic activity of*L. lactis*NZ9000 carrying pNZ45subC**

***Concentration of nisinA used for induction (ng mL***^***-1***^***)***	***Proteolytic activity (U/L)***
0.1	25.5 ± 5.9
0.5	56.7 ± 16.6
1.0	142.7 ± 6.8
5.0	73.7 ± 3.4
10.0	72.6 ± 2.0

Samples were taken in 24 hours after induction.

**Table 3:** **The proteolytic activity of*L. lactis*NZ9000 carrying pNZ45subC**

***Dilutions of NZ9700 supernatant used for induction***	***Proteolytic activity (U/L)***
Non induced	0.84 ± 0.1
100 ×	3.0 ± 0.3
1 000 ×	76.1 ± 5.9
10 000 ×	148.5 ± 0.7
20 000 ×	146.9 ± 5.4
40 000 ×	105.0 ± 2.8
60 000 ×	128.1 ± 1.8
100 000 ×	28.5 ± 0.8
200 000 ×	8.9 ± 3.9

Samples were taken in 24 hours after induction.

Up to now, many reports have described studies of protein production using NICE expression systems induced at the midlog growth phase or at high OD, however, none of them tested the induction at start point (Mierau et al. 2005a; Maischberger et al. 2010). Therefore, to simplify protein expression, we have compared the proteolytic activity of samples induced during inoculation at OD_600_ 0.1 with induction at OD_600_ 0.7 (data not shown). Interestingly, we have observed similar activity, when the cultures were induced at OD_600_ 0.7, however, only in samples supplemented with 10% milk. Most likely, the presence of calcium in milk contributes to the stability of secreted SubC. Interestingly, after a long time in extracellular environment (up to 48 h at 30°C), SubC remained stable and was active. Moreover, an induction with NZ9700 supernatant at the start point allows for simpler use of the NICE system at industrial scale.

### Signal peptide – extracellular expression

**Figure 1:**
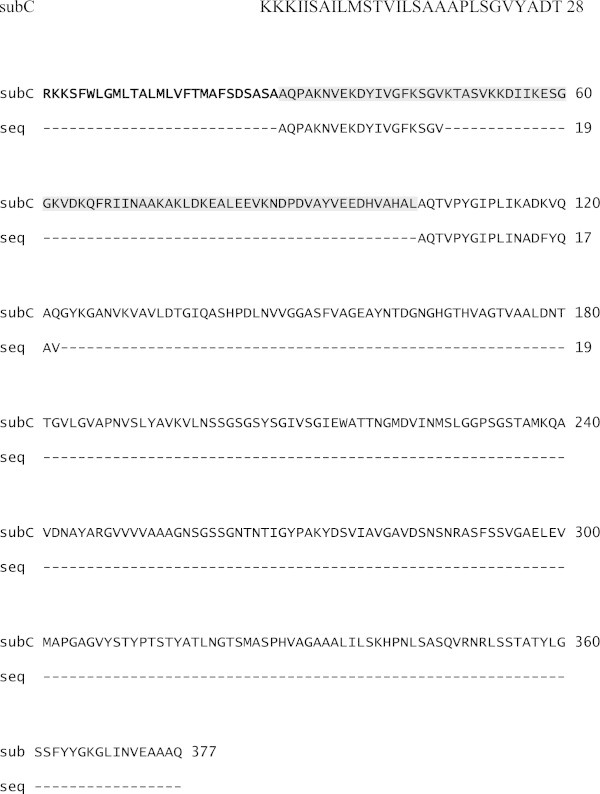
**The two 19-aa sequences obtained with Edman degradation (“seq”) compared to the subtilisin C sequence with signal peptide usp45 (“subC”).** Underlined – signal peptide usp45; bold – native signal peptide from *B. licheniformis*; grey-coloured – proregion.

The same experiment for the pNZ48subC (construct lacking SP Usp45) was performed. Noticeably, the enzymatic activity of *L. lactis* strain lacking the signal peptide was around three times lower than that observed for the pNZ45subC strain (see Table 4). As mentioned before, SubC is an extracellular protease, which possesses its own signal peptide, and is secreted via Sec machinery in *B. licheniformis. In silico* analysis showed a high homology of SeC transporters *L. lactis* and *B. licheniformis*: 74% for SecA, 64% for SecE and 67% for SecY. This explains the high level of active protease in the medium, however it is still lower in comparison with construct having native *lactococcal* signal peptide. To confirm this hypothesis SubC isolated from the medium was sequenced. The amino acids sequencing showed the presence of two subtilisins forms: immature (prosubtilisin) and mature (Figure 1). Thus, both signal peptides were properly cleaved during secretion, although signal peptide from *Bacillus* resulted in lower accumulation of protease in the extracellular medium. Therefore, for the further experiments *L. lactis* carrying pNZ45subC was used.

**Table 4:** **The proteolytic activity of*L. lactis*NZ9000 carrying pNZ45subC or pNZ48subC**

***L. lactis vectors***	***Proteolytic activity (U/L)***		
	***Non induced***	***nisinA 1ng/ml***	***10 000 × diluted NZ9700 supernatant***
pNZ45subC	0.84 ± 0.2	142.7 ± 6.7	148.5 ± 0.7
pNZ48subC	0.84 ± 0.1	46.9 ± 16.3	41.6 ± 8.1

Samples were taken in 24 hours after induction.

### Medium preferences

**Figure 2:**
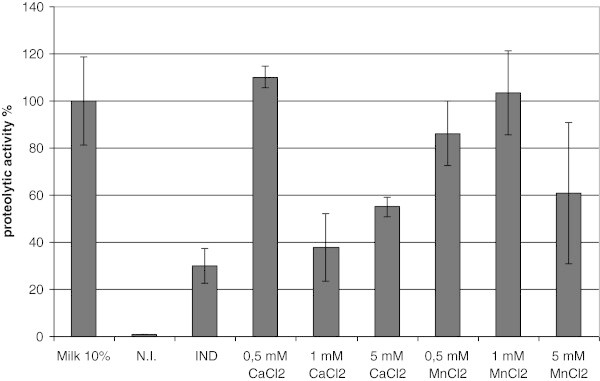
**The proteolytic activity of*L. lactis*carrying pNZ45subC tested in 24 hours after induction.** Strains were grown in GM17 medium, supplemented as indicated in the figure. The samples were induced at OD_600_ 0.1 with 10 000 NZ9700 supernatant. All data are mean values of three independent experiments; error bars indicate standard deviation.

**Figure 3:**
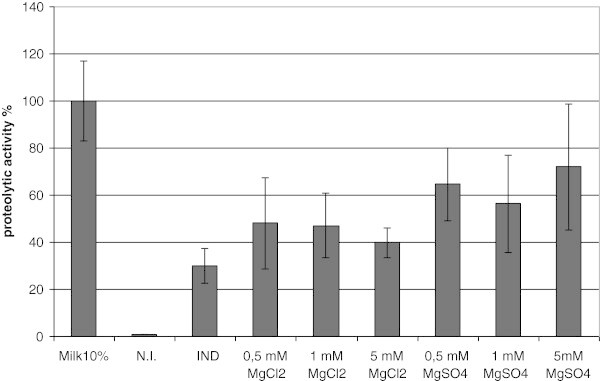
**The effect of magnesium on the SubC proteolytic activity.*L. lactis*pNZ45subC grown in GM17 supplemented with chloramphenicol and magnesium compounds as mentioned on the picture.** Samples were induced at OD_600_ 0.1 with 10 000 NZ9700 supernatant. The samples were taken in 24 hours after induction.

Proteolytic assays were performed for the strains grown on GM17 and GM17 supplemented with 10% milk (both supplemented with chloramphenicol). The highest proteolytic activity was observed during the growth on GM17 with 10% milk. The growth on simple GM17 did not lead to the highest enzymatic activity of the culture under induction (see Figure 2), which might suggest that the secreted protease is not stable or not active under these conditions. SubC possesses two calcium binding sites assisting enzyme stability (Briedigkeit & Frömmel 1989). The presence of milk in the medium could cause difficulties during the purification process of the enzyme on an industrial scale in the future. Therefore milk was replaced by different elements that are known to be present in it. To verify this thesis, different concentrations (0.5 mM - 5 mM) of CaCl_2_, MnCl_2_, MgCl_2_ and MgSO_4_ were added to GM17. The samples were collected under the same conditions as described for milk; subsequently the proteolytic assays were performed. The results are presented in the Figure 2 and Figure 3.

**Figure 4:**
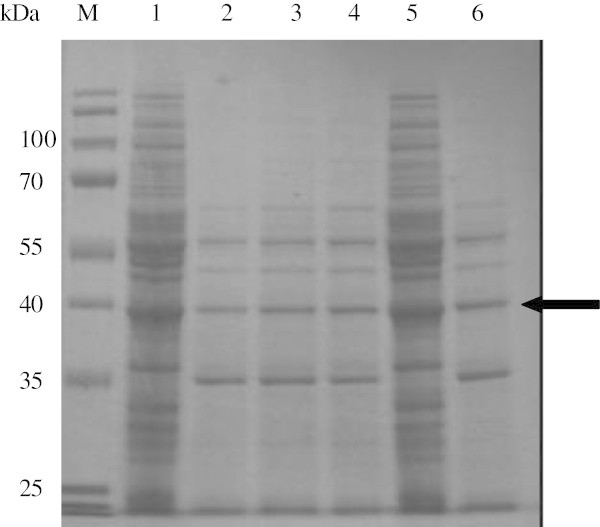
**SDS-PAGE, supernatant of*L. lactis*NZ9000 carrying pNZ45subC plasmid containing*subC*gene.** Samples were induced at OD_600_ 0.1 with 10 000 NZ9700 supernatant. GM17 media were supplemented as described below. Lane M PageRuler™ Prestained Protein Ladder (Fermentas). Lane 1 0.5 mM CaCl_2_ without induction. Lane 2 0.5 mM CaCl_2_. Lane 3 0.5 mM MgCl_2_. Lane 4 0.5 mM MnCl_2_. Lane 5 GM17 without supplements, without induction. Lane 6 GM17 without supplements. Arrow indicates bands in induced samples representing the SubC protease.

Interestingly, data showed that adding into the medium two positive ions results in an enhanced level of proteolytic activity in comparison with GM17 without supplements; however for magnesium and manganese the activity is lower than in GM17 medium supplemented with 10% milk. Moreover, the highest proteolytic activity was obtained in a medium supplemented with 0.5 mM CaCl_2_. The activity was about 10% higher than control supplemented with milk. To verify if the results obtained were caused by different amounts of protein retained in the medium, the samples of supernatants were collected and visualized on the SDS-PAGE gel (Figure 4). As is shown on the picture, non-induced samples possess many additional bands (residues from medium), while in all induced samples the number of bands is limited, this is most probably the effect of the protease activity. The expected size of SubC is 39 kDa, the corresponding band is present in all induced samples. Noticeably, the yield of the SubC protein in all induced samples seems to be the same. This data suggests that protein is produced and secreted, however measured activity depends mostly on calcium.

Next, different media that were previously shown to give higher yields of protein expressed by *L. lactis* (Mierau et al. 2005a; Berlec & Strukelj 2009) were tested for SubC production. The same experiment as described before was performed for new media. None of them showed higher proteolytic activity despite supplementing with CaCl_2_ (data not shown).

### Extension of the time of growth

The target of this study was to increase the production of secretion protein by modification of growth conditions and the parameters involved in the NICE system. Therefore, the next step of this study was verification of the protein expression (proteolitic activity) of *L. lactis* pNZ45subC and subsequently supplementation with an additional source of nutrients. Since it was shown that adding lactose as a carbon source did not significantly increased protein yield (Mierau et al. 2005a), first we decided to measure the rate of glucose utilization by *L. lactis* during the growth. Measuring glucose concentration, we have found that most of 0.5% glucose is utilized within a few hours and consequently the growth of cells slows down (data not shown). Therefore, we decided to supply additional nutrients, glucose as a source of carbon and casitone as a source of nitrogen, in the end of logarithmic phase growth. To this end, the overnight culture *L. lactis* pNZ45subC was inoculated into fresh GM17 (the start OD_600_ 0.1) medium supplemented with 0.5% glucose, 5 μg mL^-1^ chloramphenicol, 0.5 mM CaCl_2_, and 10 000 diluted NZ9700 supernatant. After 4 hours, the cultures were supplemented with glucose (0.5%) and casitone (0.1%). As a control, the induced *L. lactis* pNZ45subC culture without additional nutrients was used. The proteolytic activity was measured 4, 6 and 24 hours after induction. Surprisingly, we did not observe differences in proteolytic activity between cultures (data not shown).

**Figure 5:**
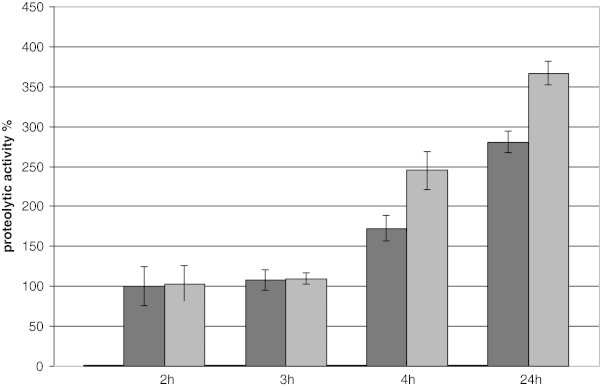
**The proteolytic activity of*L. lactis*carrying pNZ45subC.** The cultures were grown in GM17 medium supplemented with 0.5 mM CaCl_2_ and 5 μg mL^-1^ of chloramphenicol. The cultures were induced at OD_600_ 0.7. The samples were taken as indicated on the picture: 2, 3, 4 and 24 hours after induction with 10 000 NZ9700 supernatant. Pale gray bars- strain without induction (values below 1). Black bars- control strain without additional nutrients. Dark gray bars- the strain supplemented with an additional nutrients in two hours after induction. All data are mean values of three independent experiments; error bars indicate standard deviation.

Subsequently, the same experiment for the culture induced at the midlog phase growth was performed. The culture was grown under the same conditions as described before. When the culture pNZ45subC reached OD_600_ 0.6, it was induced with 10 000 diluted NZ97000 supernatant. Two hours after induction, the culture was supplemented with glucose and casitone to final concentrations 0.5%, 0.1%, respectively. As a control the induced *L. lactis* pNZ45subC culture without additional nutrients was used. The proteolytic activity was measured 2, 3, 4 and 24 hours after induction. The results are shown in Figure 5. Interestingly, the activity 2 h after induction was on the same level in both cultures; however, already in the next 2 hours it increased in the culture supplemented with additional nutrients. According to previous experiments, the highest activity was observed after 24 hours. Moreover, the activity in the supplemented culture was 30-40% higher than in the control.

**Figure 6:**
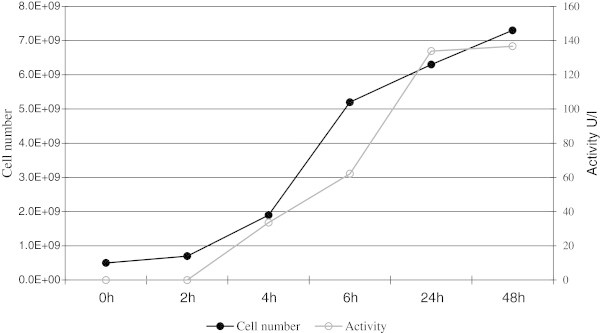
**The proteolytic activity of*L. lactis*carrying pNZ45subC in 5 L bioreactor (working volume 1.5 L).** The culture was grown in GM17 medium supplemented with 10% milk and 5 μg mL^-1^ of chloramphenicol. The culture was induced at OD_600_ 0.2. The samples were taken as indicated on the picture.

Next, we wanted to determine if our system for heterologous secreted protein production might be employed at a larger scale. Therefore an experiment with a 1.5 L batch fermentation was performed. The strain was grown in GM17 medium supplemented with 10% milk and 5 μg mL^-1^ chloramphenicol. We established pH, concentration of glucose, fermentation temperature, the time point of induction and the nisin concentration necessary for optimal SubC induction. The results obtained in bioreactor confirmed the experiments performed at the laboratory scale. *L. lactis* carrying pNZ45subC plasmid was induced at the inoculation point with the supernatant of NZ9700. Again, the highest proteolytic activity was observed 24 hours after induction, moreover, it remained stable in next coming 24 hours (Figure 6). The addition of extra nutrients to the culture induced at the start point did not increased proteolytic activity of the supernatant. In summary, the production of SubC was increased and the secreted protein remains stable, which simplifies the purification of this protease.

## Discussion

Gram-positive GRAS bacteria are recently investigated to replace host organisms such as *Escherichia coli*, for some pharmaceutical or food grade proteins production. Although different *Bacillus* species are employed in the industry they might cause problems in the production process, such as spore formation or in some strains toxins production (Pedersen et al. 2002; Salkinoja-Salonen et al. 1999). Therefore, a good option for food-relevant proteins production is lactic acid bacteria (LAB) such as *Lactococcus lactis* (Hugenholtz 2008). They are widely used in industrial fermentations, so much information is available about nutrient requirements, growth conditions etc. Moreover, for *L. lactis* the genome sequence has been published (Wegmann et al. 2007) and many genetic tools have been developed for LAB, which simplifies the usage of these microorganisms as a cell factory. Additionally they do not require aeration and only very limited mixing that significantly reduces production and reactor costs. One of the most popular expression systems in *L. lactis* is the NICE system (Mierau & Kleerebezem 2005; Kuipers et al. 1995).

In this study, we present the modification of the NICE system for heterologous secretion protease production in *L. lactis*, with possible usage at industrial scale. We used two different NICE vectors; we compared different ranges of nisinA concentration for induction and nutrient requirements. For that purpose, the *subC* gene encoding *B. licheniformis* extracellular alkaline protease was cloned downstream of the strong inducible promoter nisA and ranges of diluted supernatant of NZ9700 *L. lactis* (nisin producer) were used for the induction of secreted enzyme production. In this study, we optimized the growth and NICE- related parameters. Strikingly, laboratory strains of *L. lactis* possess only one exported housekeeping protease, HtrA involved in protein quality control at the cell surface. Moreover, HtrA is responsible for clearing anomalous proteins from the surface, and is induced under several stress conditions (Morello et al. 2008).

The expression of heterologous protease might be lethal for *L. lactis*, however it was recently shown that the use of SP Usp45 also allows the secretion of bacteriocins which are toxic for cells (Borrero et al. 2009). Therefore to avoid toxicity we added signal peptide of Usp45 to secrete SubC via Sec transporters. Indeed higher protease expression and accumulation within the cell resulted in growth inhibition (data not shown). Here we observed that the addition of *lactococcal* SP usp45 to the native signal peptide of SubC leads to better secretion, and more importantly, secreted protein remains stable, since both signal peptides are properly cleaved during protein translocation to the medium. Interestingly, a construct with two signal peptides results in higher accumulation of the protease in extracellular medium.

Subsequently, we investigated the media preferences for optimal protein production. The previously described medium (Mierau et al. 2005a) did not increase the protein level. In contrast, medium supplementation with calcium chloride resulted in superior activity of protease in the extracellular environment. Interestingly, we have also noticed that supplementing GM17 medium with other two positive ions, results in elevated proteolytic activity of SubC producer, nonetheless the highest activity was observed in samples supplemented with 0.5 mM CaCl_2_. After establishing the medium preferences for SubC production, we tested various induction time points. Hitherto, the strains carrying overexpression constructs were induced at the midlog growth phase or at high OD (Mierau et al. 2005a; Maischberger et al. 2010) although we have not confirmed the reports showing the results of induction at start point. To this end, the overnight cultures of *L. lactis* carrying pNZ45subC or pNZ48subC were inoculated into fresh medium, supplemented as aforementioned, and induced at OD_600_ 0.1 and 0.6 and with various ranges of NZ9700 supernatant. The samples were tested for proteolytic activity 24 hours after induction. We did not observe significant differences in the proteolytic activity between all samples, which might suggest that the level of functional SubC protease was the same in all cultures. In contrast to other reports (Berlec et al. 2008), we did not observe a correlation between increasing OD and protein production. One of the most interesting parts of this phenomenon is the problem of the stability/activity of SubC in the extracellular environment. Strikingly, the SDS-PAGE data showed that the level of protein production in various cultures was unchanged; however the proteolytic activity was different. This could result from various proportions of mature versus immature protease as seen from SubC amino acid sequence determination. Noticeably, the overproduction of protein might also cause stress responses and consequently degradation of the target protein in the cells (Thumm & Gotz 1997).

We compared different nisinA concentrations for *L. lactis* induction. The studied nisin concentration range was from 0.1 to 10.0 ng mL^-1^ and the highest proteolytic activity was obtained when 1 ng mL^-1^ of nisinA or 10 000 – 20 000 diluted supernatant of *L. lactis* NZ9700 was used. In comparison, nisin concentration in published data varies from 0.5 to 40 ng mL^-1^ (Mierau et al. 2005b). Thus induction during inoculation of the bioreactor enables reduction of the inducer amount and simplifies the production process. One of the most interesting issues of this study was an extension of the logarithmic phase growth of *L. lactis*, since during this phase the highest activity was observed.

Initial experiments showed that *L. lactis* utilizes most of the 0.5% glucose present in GM17 medium. Consequently, the next step was to supply more nitrogen and carbon sources. The cultures were induced at OD_600_ 0.6, the additional nutrients were added in two hours after induction. Interestingly, we observed increased proteolytic activity already 2 hours after supplementing with nutrients. The differences between the target strain and the control were between 30-40%, the highest differences were observed in 6 hours after induction, and remained stable up to 24^th^ hour after inductions.

In summary, we optimized the NICE expression system for heterologous secretion protease production. The use of a GRAS expression host for secreted enzyme production will make it possible to use these proteins much more easily and economically, than in the case of intracellular production. The described expression system might be used for industrial production of rennet or direct application of such strain in dairy.
